# Root exudation rate as functional trait involved in plant nutrient‐use strategy classification

**DOI:** 10.1002/ece3.4383

**Published:** 2018-07-30

**Authors:** Julien P. Guyonnet, Amélie A. M. Cantarel, Laurent Simon, Feth el Zahar Haichar

**Affiliations:** ^1^ UMR CNRS 5557 Laboratoire d'Ecologie Microbienne UMR INRA 1418 Univ Lyon Université Claude Bernard Lyon 1 University of Lyon Villeurbanne Cedex France; ^2^ UMR5023 LEHNA Université Lyon 1 CNRS ENTPE Univ Lyon Université Claude Bernard Lyon 1 University of Lyon Villeurbanne Cedex France

**Keywords:** conservative strategy, exploitative strategy, plant functional trait, plant resource‐use strategies, rhizosphere, root exudation level

## Abstract

Plants adopt a variety of life history strategies to succeed in the Earth's diverse environments. Using functional traits which are defined as “morphological, biochemical, physiological, or phonological” characteristics measurable at the individual level, plants are classified according to their species’ adaptative strategies, more than their taxonomy, from fast growing plant species to slower‐growing conservative species. These different strategies probably influence the input and output of carbon (C)‐resources, from the assimilation of carbon by photosynthesis to its release in the rhizosphere soil via root exudation. However, while root exudation was known to mediate plant‐microbe interactions in the rhizosphere, it was not used as functional trait until recently. Here, we assess whether root exudate levels are useful plant functional traits in the classification of plant nutrient‐use strategies and classical trait syndromes? For this purpose, we conducted an experiment with six grass species representing along a gradient of plant resource‐use strategies, from conservative species, characterized by low biomass nitrogen (N) concentrations and a long lifespans, to exploitative species, characterized by high rates of photosynthesis and rapid rates of N acquisition. Leaf and root traits were measured for each grass and root exudate rate for each planted soil sample. Classical trait syndromes in plant ecology were found for leaf and root traits, with negative relationships observed between specific leaf area and leaf dry matter content or between specific root length and root dry matter content. However, a new root trait syndrome was also found with root exudation levels correlating with plant resource‐use strategy patterns, specifically, between root exudation rate and root dry matter content. We therefore propose root exudation rate can be used as a key functional trait in plant ecology studies and plant strategy classification.

## 1 INTRODUCTION

Plants are able to colonize a broad diversity of environments due to a variety of different strategies to manage nutrient acquisition, use and conservation. These resource‐use strategies can be explained by physiological characteristics which are measurable as plant functional traits, defined as “morphological or physiological characteristics measurable at the individual level, from the cell to the whole‐organism level, without reference to the environment or any other level of organization which impact fitness indirectly via their effects on growth, reproduction and survival” (McGill, Enquist, Weiher, & Westoby, 2006; Violle et al., 2007). Grass species (i.e. *Poaceae*) are monocotyledon pioneer plants colonizing a large part of open landscapes, due to a wide variety of functional traits such as those described by Fort, Jouany, and Cruz (2013) and Grigulis et al. (2013). Functional traits are used to classify plant species according to their growth performance. The “leaf economics spectrum” (Wright et al., 2004) describes such relationships by highlighting the link between the specific leaf area (SLA) or the leaf nitrogen concentration (LNC), and a spectrum ranging from fast‐growing plant species (i.e. exploitative species) with higher photosynthetic capacity and rapid rates of N acquisition (Aerts & Chapin, 1999), to slow‐growing plant species (i.e. conservatives species) associated with the conservation of nutrients in thick leaf tissues (Aerts & Chapin, 1999; De Deyn, Cornelissen, & Bardgett, 2008; Osnas, Lichstein, Reich, & Pacala, 2013; Personeni & Loiseau, 2004; Reich, 2014; Wright et al., 2004). These trade‐offs between leaf acquisition and conservation of resources has also been suggested to occur for root traits (Birouste, Kazakou, Blanchard, & Roumet, 2012; Fort et al., 2013; Mommer & Weemstra, 2012; Roumet, Urcelay, & Dıaz, 2006), and recent studies demonstrated the value of root traits as indicators of ecosystem functions (Grassein et al., 2015; Roumet et al., 2016) and soil functions (Cantarel et al., 2015; Moreau et al., 2015). These results suggest that the plant strategies of nutrient management strongly influence the carbon (C)‐resources input and output, from the assimilation of carbon by photosynthesis to its release in the rhizosphere soil via root exudation.

Root exudation is a key process for carbon (C) transfer into the soil and can release up to 20% of carbon fixed by photosynthesis by root exudation (Haichar et al., 2008). These root exudates, defined as primary and secondary metabolites, represent a source of nutrients for microorganisms, and play a key role in the establishment of plant‐microorganisms interactions (Bais, Weir, Perry, Gilroy, & Vivanco, 2006; Guyonnet et al., 2017; Haichar, Fochesato, & Achouak, 2013; Haichar, Santaella, Heulin, & Achouak, 2014; Philippot, Raaijmakers, Lemanceau, & van der Putten, 2013). However, plant root exudation is poorly understood relative to other processes in the terrestrial C cycle (Jones, Hodge, & Kuzyakov, 2004). In addition, a possible difference in root exudation between competitive and conservative plants has been suggested (De Deyn et al., 2008; Personeni & Loiseau, 2004). However, to the best of our knowledge, there are currently no data available that directly compares root exudation levels from exploitative and conservative species in connection to plant functional traits used to classify plant according to their nutrient‐use strategies.

The aim of this work was to investigate the link between plant functional traits and root exudation rate, and how this could affect plant resource‐use strategy classification. This study is based on the hypothesis that exploitative plants, characterized by high SLA and specific root length (SRL), produce more root exudates than conservative plants, characterized by low SRL and SLA and high LDMC and RDMC. To test this hypothesis, six grasses were selected that represent a resource‐use strategy gradient, and were cultivated in the same soil for 11 weeks. Plant functional traits and root exudation rate were measured for each plant's rhizosphere after one week of ^13^CO_2_ labeling.

## 2 MATERIALS AND METHODS

### 2.1 Plant growth

A laboratory experiment was performed using six perennial C3 grass species representing a gradient of plant nutrient use strategies (Cantarel et al., 2015; Gross et al., 2009; Maire, Gross, Da Silveira Pontes, Picon‐Cochard, & Soussana, 2009): two conservative (*Festuca paniculata*, FP and *Sesleria caerulea,* SC), two intermediate (*Bromus erectus*, BE and *Anthoxanthum odoratum,* AO) and two exploitative (*Dactylis glomerata*, DG and *Trisetum flavescens*, TF). All six grasses belong to the *Pooideae* subfamily (*Pooideae* phylogeny and classification of six grasses shown in Supporting Information Figure S1). Grass species were collected in the French Alps (Lautaret Pass; 45°2′5.1″N, 6°22′43.5″E; Elevation: 2,000 masl), sampled and separated into individual tillers in the field. For each plant species, seven tillers (i.e. seven clones by species) with three young mature leaves and the root system clipped to 3 cm were grown under controlled conditions to standardize plant growth. Plants were cultivated on a luvisol soil with no added nitrogen source collected from La Côte Saint‐André (Isère, France), which is continuously cropped with maize (Guyonnet et al., 2017). The soil pH (7.7) was measured following ISO 10390 before and after plant culture, with plant growth having no effect on soil pH. The soil was sieved (2 mm mesh size) and 170 g were placed into plastic pots. The seven individuals from each plant species were grown in a greenhouse (13 hr day 22°C/11 hr night 18°C) with a light intensity of approximately 8–10 klux for 11 weeks. Soil was moistened by immersion in water every 3 days. Four pots with bulk soil (i.e. without plants) were treated as controls under the same conditions.

### 2.2 Plant ^13^C‐labelling

Continuous labelling was initiated 10 weeks after plant growth according to Haichar et al. (2008). Plants were placed in a growth chamber (developed and managed by “Groupe de Recherche Appliquées en Phytotechnologies,” CEA Cadarache, France) equipped for automatic control of light, temperature, moisture, evapotranspiration, irrigation and CO_2_ concentration. The day–night cycle was set at 8/16, respectively, with a light intensity of 13.5 klux, maximum daily temperatures ranging from 20 to 22°C, air moisture was adjusted to 80% and CO_2_ concentration was maintained at 350 μl/L. CO_2_ partial pressure was kept constant by injection of ^13^CO_2_ (>99% atom ^13^C; purchased from Cortec Net, Paris, France) during active photosynthesis. The isotope excess of CO_2_ and the partial pressure in the chamber were continuously monitored by near infra‐red spectroscopy. To avoid dilution of ^13^CO_2_ by ^12^CO_2_ from soil respiration, the CO_2_ concentration of the chamber was lowered to 300 μl/L at the end of the night period by automatic gas trapping using NaOH. ^13^CO_2_ was then injected to give a CO_2_ concentration of 350 μl/L. The isotope excess in the chamber was maintained at > 95% atom ^13^C during the 7 days of labelling. Plants and bulk soil (BS) were collected after ^13^CO_2_ labeling.

### 2.3 Plant harvesting

At the end of labelling experiment, four individuals from each plant population were used to measure functional traits and quantify root exudation. Immediately following plant harvest, four tiller bases from each plant species were placed in de‐ionized water and then stored at 4°C in the dark for at least 6 hr to allow full leaf rehydration (Garnier, Shipley, Roumet, & Laurent, 2001). The roots of each plant were separated manually from the root‐adhering soil (RAS), corresponding to the immediate environment where plants take up water and nutrients for their growth (Alami, Achouak, Marol, & Heulin, 2000). The roots were washed carefully with distilled water to remove any remaining soil particles and then placed in distilled water and stored at 4°C. RAS was separated carefully from the remaining fine roots in order to measure ^13^C enrichment.

### 2.4 Plant functional trait measurements

#### 2.4.1 Leaf traits

Specific leaf area, expressed as the ratio of leaf area to leaf dry mass (cm^2^/g), and leaf dry matter content (LDMC), expressed as the ratio of leaf dry mass and leaf saturated fresh mass (mg/g) were measured following standard protocols (Cornelissen et al., 2003). Briefly, after rehydration, the lamina of the youngest fully expanded leaf of each replicate was measured, weighed and its area determined using WINFOLIA software (Epson Perfection V700 PHOTO, Nagano, Japan). Leaf aliquots were then oven‐dried at 105°C for 48 hr and weighed with a precision balance.

N concentration and C‐stable isotope composition were measured using an isotope ratio mass spectrometer coupled with an elemental analyser (EA‐IRMS; Isoprime 100, Isoprime Ltd, UK and Thermo FlashEA 1112, ThermoElectron, USA). Measurements were made using 1 mg samples of lyophilized, ground plant tissue weighed into tin capsules (Elemental Microanalysis, UK). N concentrations were calibrated using aspartic acid (10.52% N) as a reference material. For ^13^C/^12^C measurements, IAEA‐CH3, IAEA CH6 reference materials and ^13^C glucose (10%) were analysed with the samples. C isotope compositions were expressed as δ in ‰ with V‐PDB as standard.

#### 2.4.2 Root traits

Fresh roots were scanned (Epson Perfection V700 PHOTO, Nagano, Japan) and analysed with WINRHIZO software to determine root length. SRL (cm/g) is the ratio of root length (cm) to mass (g). The roots were then oven‐dried at 105°C for 48 hr and weighed. Root dry matter content (RDMC, mg/g) is the oven dry mass (mg) of root divided by its water‐saturated fresh mass (g). Root nitrogen content (RNC, %) was measured using the same protocol as for leaf N content (LNC).

#### 2.4.3 Root exudation

Root exudates levels were measured by quantifying ^13^C enrichment in the root‐adhering soil, assuming that all of the ^13^C increase above natural abundance was derived mainly from exudates produced by ^13^C labelled plants. Aliquots of adherent soil were lyophilized and 8 mg of RAS were placed into 5 × 8 mm “Ultra Clean” tin capsules and analysed using EA‐IRMS.

To determine the ^13^C natural abundance in soil and plants, triplicate grass samples were cultivated without ^13^C‐labeling during the 11 weeks period. Unlabelled plant and soil were harvested and analysed with the same protocols as described above. The ^13^C concentration in RAS was determined using the equation:

$$\left[{}^{13}C\right]=\left(\delta {}^{13}C-\delta {{}^{13}C}_{\mathrm{control}}\right)\times \frac{R_{\mathrm{standard}}}{10^{3}}\times \left[C\right]$$

where [^13^C] estimates the concentration of exudates in soil or assimilation by microorganisms present into the soil, in mg of ^13^C per kg of soil (equivalent dry weight). δ^13^C is the C isotope composition of the soil organic carbon, δ^13^C_control_ the C isotope composition of the soil organic carbon, *R*_standard_ the ^13^C/^12^C of V‐PDB (0.0112372) and [C] the carbon concentration of the soil.

### 2.5 Statistical analysis

A principal component analysis (PCA, R package FactorMineR 1.29) was used to explore the distribution of plants relative to their resource‐use strategies and plant functional traits, particularly for identifying specific plant traits explaining resource‐use strategies. Correlations between functional traits were tested using a Pearson test for normal data or a Spearman test for nonnormal data.

Effects with a *p*‐value <0.05 are referred to as significant. All statistical analyses were carried out using R 3.1.2.

## 3 RESULTS

### 3.1 Leaf and root trait syndromes

**Figure 1:**
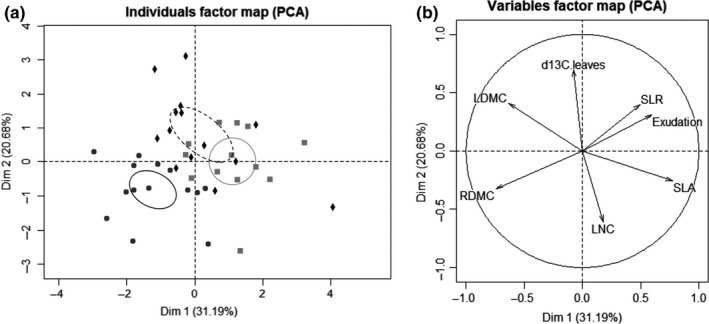
Principal component analysis ordination of the distribution of six grasses based on resource‐use strategies, illustrated by plant functional traits. (a) Points show the scores of all individual species as a function of their strategies: conservative (●), intermediate (♦) and exploitative (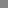) and (b) solid lines show the loading of plant traits (LDMC: leaf dry mass content; LNC: leaf nitrogen content; RDMC: roots dry mass content; RNC: root nitrogen content and root exudation; SLA: specific leaf area; SLR: specific length root; δ^13^C leaf: photosynthesis efficiency)

Plant resource strategies were characterized using different leaf and root functional traits. Some plant traits, such as leaf dry mass content (LDMC), δ^13^C values of leaves and root nitrogen content (RNC), were not significantly different between grasses (Table 1). However, root dry mass content (RDMC) was significantly different between *T. flavescens* (TF) and conservative grasses (*S. caerulea* [SC] and *F. paniculata* [FP]; Table 1). The species encompassed a wide spectrum of variability for others functional traits (Table 1), such as SLA (*F*_5_ = 28.1, *p* < 0.007), SRL (*F*_5_ = 9.2, *p* < 0.004) and leaf nitrogen content (LNC; *F*_5_ = 11.5, *p* < 3.10^−4^). These three functional traits were the most variable traits between grass species and between plant resource strategies. Leaf and root traits provided a good separation of our plant species along a gradient of plant resource‐use strategies, with conservative grasses (*S. caerulea* (SC) and *F. paniculata* (FP) at one end, and a range of exploitative grasses on the other end (*D. glomerata* [DG], *B. erectus* [BE], *A. odoratum* [AO] and *T. flavescens* [TF]; Figure 1a).

**Table 1:** Plant functional traits measured for six perennial grasses representing a gradient of resource‐use strategies

Species	SLA (cm^2^/g)	LDMC (mg/g)	SRL (cm/g)	RDMC (mg/g)	δ^13^C leafs (‰)	LNC (%)	RNC (%)	Exudation (μg/kg)
*Sesleria caerulea*	224.70 ± 17.38^b^	3.16E‐04 ± 3.58E‐05^a^	7067.29 ± 1976.48^b^	7.34E‐04 ± 7.83E‐05^a^	10416.88 ± 2235.41^a^	2.08 ± 0.16^a^	0.97 ± 0.16^a^	15.02 ± 10.07^b^
*Festuca paniculata*	138.09 ± 5.91^a^	3.06E‐04 ± 1.45E‐05^a^	8893.82 ± 1918.14^b^	7.14E‐04 ± 3.19E‐05^a^	8334.66 ± 1483.52^a^	1.83 ± 0.21^a^	1.00 ± 0.21^a^	17.74 ± 12.26^b^
*Bromus erectus*	199.37 ± 23.00^b^	2.82E‐04 ± 1.36E‐05^a^	15622.36 ± 2582.25^b^	5.01E‐04 ± 3.76E‐05^a^	17266.10 ± 5333.43^a^	1.33 ± 0.14^b^	0.95 ± 0.04^a^	97.90 ± 7.68^a^
*Anthoxanthum odoratum*	370.47 ± 68.00^b^	2.96E‐04 ± 2.47E‐05^a^	18262.44 ± 2904.37^ab^	5.51E‐04 ± 4.58E‐05^a^	17760.82 ± 2878.25^a^	1.21 ± 0.06^b^	1.00 ± 0.06^a^	33.39 ± 11.41^ab^
*Dactylis glomerata*	240.18 ± 9.43^b^	3.14E‐04 ± 1.53E‐05^a^	33658.14 ± 5591.74^a^	5.65E‐04 ± 9.51E‐05^a^	7615.75 ± 4331.97^a^	1.12 ± 0.04^b^	1.04 ± 0.14^a^	49.83 ± 5.04^ab^
*Trisetum flavescens*	472.08 ± 22.83^c^	2.75E‐04 ± 7.84E‐06^a^	16262.39 ± 1455.30^ab^	4.71E‐04 ± 8.07E‐05^a^	10230.39 ± 2009.41^a^	2.18 ± 0.07^a^	0.90 ± 0.07^a^	111.21 ± 31.66^a^
*p*‐value	0.007	0.2	0.004	0.01	0.07	3 × 10^−4^	0.8	0.04

Notes

Data are means ± standard errors (*SE*).

Plant traits are labelled as LDMC: leaf dry mass content; LNC: leaf nitrogen content; RDMC: roots dry mass content; RNC: root nitrogen content and root exudation; SLA: specific leaf area; SLR: specific length root; δ^13^C leaf: photosynthesis efficiency.

Values followed by the same letter are not significantly different according to ANOVA or Kruskal–Wallis tests (*p* < 0.05).

**Figure 2:**
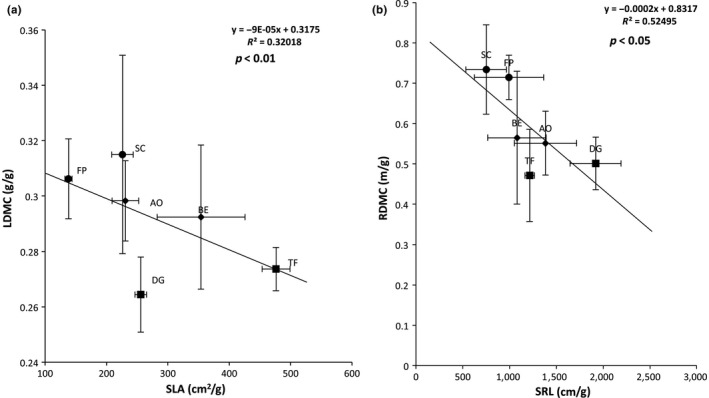
Relationship between (a) LDMC and SLA, and (b) RDMC and SRL of six grass species representing a gradient of resource‐use strategies: *Sesleria caerulea* (SC); *Festuca paniculata*, (FP); *Anthoxanthum odoratum* (AO), *Bromus erectus* (BE); *Trisetum flavescens* (TF) and *Dactylis glomerata* (DG). Values and error bars represent the mean and standard errors of 4 replicates values, respectively. *p* values <0.05 represents significant correlation

Grasses were distributed along a RDMC‐SRL axis on axis 1 (Figure 1b) of the PCA and described plant nutrient conservation axis. Axis 2 was driven by leaf δ^13^C and LNC, which represented plant photosynthesis efficiency or plant nutrient acquisition. PCA showed a strong negative relationship between SLA and LDMC values and between SRL and RDMC values (Figure 1b). A significant negative correlation was found for SLA and LDMC (Pearson correlation = −0.40, *p* < 0.01, Figure 2a) and SRL and RDMC (Pearson correlation = −0.43, *p* = 0.05, Figure 2b). These traits (SLA, LDMC, SRL, and RDMC) were related to the plant resource strategy gradient. Plants such as SC and FP were characterized by a high LDMC and RDMC and a low SLA and SRL, typical of the conservative syndrome, whereas plants such as BE, AO, DG, and TF are characterized by a high SLA and SRL and a low LDMC and RDMC, which are traits that are typical of the exploitative syndrome.

### 3.2 Root exudation quantity as resource‐use strategy indicators

**Figure 3:**
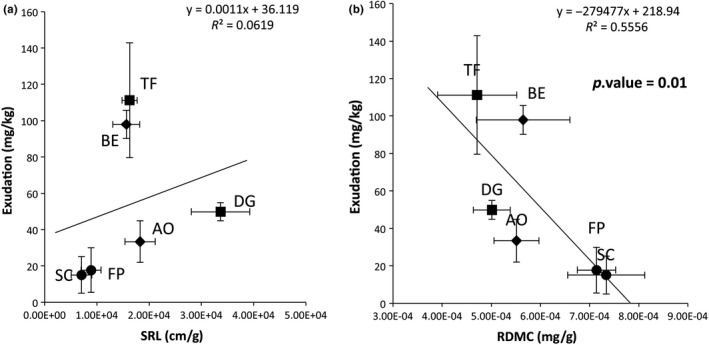
Relationship between (a) specific root length and root exudation, and (b) RDMC and root exudation of six grass species representing a gradient of resource‐use strategies: *Sesleria caerulea* (SC); *Festuca paniculata*, (FP); *Anthoxanthum odoratum* (AO), *Bromus erectus* (BE); *Trisetum flavescens* (TF) and *Dactylis glomerata* (DG). Data points are the mean of four replicates and error bars represent standard errors. *p* values <0.05 represents significant correlation

As for LNC and SLA, root exudation levels were significantly different between species (*F*_5_ = 7.4, *p* < 0.04; Table 1). Conservative grasses (SC and FP) produced less exudate than the other plants. BE and TF were the two plants which exudated the greater amount of carbon compounds (Table 1 and Figure 3). PCA (Figure 1b) indicated that root exudate levels were correlated positively with SRL (Figure 2a, Spearman correlation = 0.63, *p* = 0.03), and with RDMC negatively (Figure 1b, Spearman correlation = −0.67, *p* = 0.02), but with no significant difference for RDMC between plant species (*p* > 0.05).

## 4 DISCUSSION

Over the last 10 years, numerous studies have examined the importance of plant functional traits and are used to determine plant resource‐use strategies (Grigulis et al., 2013; Orwin et al., 2010). However, to the best of our knowledge, the relationships between leaf and root functional traits and root exudation levels remain unclear. Here, we investigated how root exudate levels could be considered as a functional marker driving plant resource‐use strategies.

### 4.1 Relationships between leaf and root traits in shaping plant resource‐use strategies

As reported in a large number of previous studies, plant functional traits are good indicators of plant resource‐use strategies (Grime, 2006; Reich et al., 2003; Wright et al., 2004). As functional traits are also aspects of phenotype variations, they could evolve along the phylogeny branches. Dıaz et al. (2013) showed that the evolutionarily closely related species tend to be ecologically similar and have close functional trait values. However, *Poaceae* are one of the largest and most diverse families in the angiosperms (Watson & Dallwitz, 1992) with a wide geographic, environmental, and ecological distribution. Due to this large environmental and ecology spectrum, closely related grass genera can have contrasting plant resource‐use strategies. For example, related phylogenetic genera such as *Lolium* and *Brachypodium* (Supporting Information Figure S1, Bouchenak‐Khelladi et al., 2008) have been shown to have different strategies, as *Lolium* sp. can be consider an exploitative species and *Brachypodium sp*., a conservative species (Fort et al., 2013).

In this study, classical trait syndromes showed negative correlations between SLA‐LDMC and SRL‐RDMC. These classical syndromes in plant ecology allow species to be classified according to their own resource‐use strategy. Thus, grasses, such as *S. caerulea* (SC) and *F. paniculata* (FP) are characterized by low SLA and SRL, and high LDMC and RDMC, characteristic of conservative plants with low rates of photosynthesis and nutrient acquisition and maintains nutrients in their tissues (Aerts & Chapin, 1999). Conversely, grasses such as *A. odoratum* (AO), *B. erectus* (BE), *D. glomerata* (DG) and *T. flavescens* (TF) are characterized by higher SLA and SRL and lower LDMC and RDMC, characteristic of exploitative plants which maximize photosynthesis and nutrient acquisition (Aerts & Chapin, 1999). According to the literature, BE and AO are classified as intermediates along the gradient of resource‐use strategies (Maire et al., 2009). Under the conditions of our study, by combining leaf and root traits, we showed that these two plant species possessed exploitative profiles. In comparison, Fort et al. (2013) classified BE as a drought tolerant plant, with conservative functional traits. This variability can be the result of plant phenotypic plasticity to colonize a broader diversity of environments, (Sultan, 2000). These findings suggest that functional traits can vary as a function of the environment, as demonstrated in previous studies by Sultan (2000) and Cornelissen et al. (2003).

### 4.2 Root exudation rate as a key functional trait

In addition to well‐documented functional traits, we investigated root exudation levels as a functional trait and a key indicator of plant resource‐use strategies. Root exudation levels allowed us to classify the six plants along a resource‐use strategy gradient with two conservative and four exploitative plants. We demonstrated that exploitative exuded more than conservative grasses, as observed by Kaštovská, Edwards, Picek, and Šantrůčková (2014). We also showed novel root trait syndromes linking the level of root exudation and root functional traits. This could be explained by the fact that plants with a high growth rate (i.e. exploitative plants) exuded more than plants with a lower grow rate (i.e. conservative plants). In addition, conservative plants are known to store their nutrients in their tissues and limit C losses (De Deyn et al., 2008; Personeni & Loiseau, 2004). The level of root exudation was correlated negatively to RDMC, a root trait involved in nutrient conservation (Roumet, Lafont, Sari, Warembourg, & Garnier, 2008). Some studies have shown that plants with a strong RDMC had a higher proportion of xylem in their root tissues (Hummel et al., 2007; Wahl & Ryser, 2000). The xylem of angiosperms can have three functions: (a) water transport, (b) mechanical support and (c) storage of water and nutrients (Pratt, Jacobsen, Ewers, & Davis, 2007). This high proportion of xylem in roots could explain why conservative plants can store nutrients in their root tissues. Moreover, root exudation was positively linked to SRL, described as “the amount of “harvesting” or absorptive root tissue deployed per unit mass invested” (Cornelissen et al., 2003), but not correlated to photosynthesis efficiency (estimated by the leaf ^13^C content), as shown by Groleau‐Renaud, Plantureux, and Guckert (1998). These authors demonstrated, under axenic hydroponic culture conditions, that exudates from maize plants were not linked to photosynthesis activity; however, root surface area was strongly linked to root exudation. This could be explained by an increase in surface exchange between roots and soil, and more apical zones, which have been shown to be the preferred exudation sites (Personeni, Nguyen, Marchal, & Pagès, 2007). These results showed the importance of root functional trait studies to understand root exudation. Hence, root exudation level, which is correlated to other root traits, could be considered as a plant physiological functional trait, and used to determine plant nutrient strategy. A low level of carbon exudate thus seems to be a functional trait characteristic of conservative plants, and a high level of carbon exudate a functional trait characteristic of exploitative species.

From this laboratory study, we can conclude that plant resource‐use strategies are linked to the carbon levels exuded into the root adhering‐soil. Future studies are needed to confirm our results on a large range of *Poaceae* species. In addition, these results need to be compared with those of other plant families before it can be generalized.

At the time point when root exudate levels were analysed, the growth stage of plant species studied here corresponded to the vegetative phase and it is well known that root exudation levels vary during the life cycle of plants (Jones, Nguyen, & Finlay, 2009) suggesting a relationship between plant physiology and root exudate level. Therefore, we believe that time‐course experiments will make it possible to test the link between root exudate level and plant nutrient‐use strategy classification. Further studies should also test this link under different growth and environmental conditions (e.g. spring vs. winter) as root exudation also varies according to different abiotic factors (Haichar et al., 2014). In conclusion, the present study is the first to consider exudation rate as a key plant functional trait to determine plant resource‐use strategies.

## CONFLICT OF INTERESTS

The authors declare that they have no competing interests.

## AVAILABILITY OF DATA AND MATERIALS

The full datasets generated and used in this current study are available from the corresponding authors upon reasonable request.

## AUTHOR CONTRIBUTIONS

FZH and AAMC designed the experiments. JPG, AAMC and FZH performed experiments. LS performed the ^13^C measurement. JPG and FZH drafted the manuscript and all authors reviewed and approved the final manuscript.
