# Supplementary material for: Root exudation rate as functional trait involved in plant nutrient‐use strategy classification
Source: Ecol Evol. 2018 Jul 30;8(16):8573–81. doi: 10.1002/ece3.4383 (PMC6144958; doi:10.1002/ece3.4383)
Supplement: Supplementary file 1 [file ECE3-8-8573-s001.docx]

**Figure S1**. A cladogram highlighting the principal *Pooideae* genera and the relationships of the 6 studied species (names in bold). Tree adapted from Bouchenak-Khelladi *et al*. 2008.

*Festuca* (***Festuca paniculata***)

*Vulpia*

*Castellia*

*Deschampsia*

*Catapodium*

*Cynosurus*

*Dactylis (****Dactylis glomerata****)*

*Holcus*

*Sesleria (****Sesleria caerulea****)*

*Alopecurus*

*Poa*

*Helicotrichon*

*Arrhenatherum*

*Avena*

*Koeleria*

*Trisetum (****Trisetum flavescens****)*

*Anthoxanthum (****Anthoxanthum odoratum****)*

*Briza*

*Phalaris*

*Elymus*

*Leymus*

*Triticum*

*Agropyron*

*Hordeum*

*Psathyrostachys*

*Bromus (****Bromus erectus****)*

*Lolium*

*Brachypodium*

*Glyceria*

*Melica*

*Stipa*
